# Tumour microenvironment of pancreatic cancer: immune landscape is dictated by molecular and histopathological features

**DOI:** 10.1038/s41416-019-0479-5

**Published:** 2019-05-21

**Authors:** Eva Karamitopoulou

**Affiliations:** Pancreatic Cancer Research Group, Division of Clinical Pathology and Translational Research Unit, Institute of Pathology, University of Bern, Murtenstrasse 31, Bern, 3008 Switzerland

**Keywords:** Prognostic markers, Predictive markers

## Abstract

Pancreatic cancer is a lethal disease, with fewer than 7% of patients surviving beyond 5 years following diagnosis. Immune responses are known to influence tumour progression. The dynamic interaction between cancer cells and immune cells in the tumour microenvironment (TME) can not only result in, or be influenced by, different tumour characteristics, but it can also lead to diverse mechanisms of immune evasion. At present, there is much interest in classifying pancreatic cancer according to its morphologic, genetic and immunologic features in order to understand the significant heterogeneity of this tumour type. Such information can contribute to the identification of highly needed novel prognostic and predictive biomarkers, and can be used for accurate patient stratification and therapy guidance. This review focuses on the characteristics of the local immune contexture of pancreatic ductal adenocarcinoma and the interaction between tumour cells and immune cells within the TME, by simultaneously taking into account the histomorphologic and genetic features of the tumours. The emerging opportunities for approaches that could predict the most-effective therapeutic modalities towards more targeted, personalised treatments to improve patient care are also discussed.

## Background

Pancreatic ductal adenocarcinoma (PDAC) is an aggressive disease that frequently presents at an advanced stage.^1^ Moreover, the incidence of PDAC is rising, and it is predicted to become the second leading cause of cancer-related death by the year 2030.^2^ Despite increased knowledge regarding its genetic background, PDAC remains refractory to most currently available treatment modalities.^3^ As a tumour type that is known to have low immunogenicity and an immunosuppresive microenvironment, the administration of cancer immunotherapy through checkpoint inhibition has, until now, shown limited success in patients with PDAC, though it has led to improved outcomes for many other cancers.^4^ Furthermore, there are currently no targeted therapies for the main driver mutations known to occur in PDAC, such as *KRAS, TP53, CDKN2A* and *SMAD4*.^5^

As the immune system is known to have a crucial role in cancer, the characterisation of the immune component of the tumour microenvironment (TME) can provide valuable information regarding the ways in which the host immune response interacts with cancer cells. This information can be employed for guiding the use of immunotherapies and other immunomodulatory approaches.^6^

This review summarises the main characteristics of the very complex and heterogeneous immune landscape of PDAC, focusing on the dynamic interplay between cancer cells and immune cells within the TME in association with the morphologic and genetic features of the tumour type. As specific immune compositions might render the TME more or less amenable to cancer cell invasion, understanding the interaction between tumour cells and immune cells should increase our knowledge of the diverse mechanisms of immune evasion employed by pancreatic cancer. Moreover, integrating molecular, morphologic and immunophenotypic findings could yield valuable clues that may not only provide an insight into the different immunosuppressive mechanisms present in PDAC, but also benefit the development of strategies for a moretargeted approach to the use of immunotherapy and/or other combinatorial treatments to augment immunity in the TME of PDAC.

## Genomic characteristics and molecular subtypes of pancreatic cancer

Recent approaches to the classification of PDAC have aimed to refine patient stratification based on genomic criteria. Sequencing studies have revealed a small number of main driver mutations (in *KRAS, TP53, CDKN2A* and *SMAD4*) that are present in different combinations in most PDACs. *KRAS* is mutated in >90% of PDACs, but even most tumours with wild-type *KRAS* contain somatic genetic alterations that activate the RAS/mitogen-activated protein kinase pathway upstream or downstream of KRAS, suggesting that the RAS pathway remains an important molecular driver of pancreatic tumorigenesis even in the absence of *KRAS* mutations.^7^ Inactivating mutations in *TP53* are the second most common genetic alteration occurring in ~70% of PDACs.^8,9^

*CDKN2A* can be downregulated through multiple mechanisms, including DNA methylation, deletions and intragenic mutation. Moreover, recent data reported by The Cancer Genome Atlas (TCGA) pancreas cancer project have revealed a disproportionate number of samples with *CDKN2A* alterations in the high neoplastic cellularity group, which underscores the fact that low neoplastic cellularity may obscure genetic alterations.^7^

*SMAD4* mutation and/or inactivation is found in >50% of invasive pancreatic adenocarcinomas. Smad4 is a member of the Smad family of signal transducers and acts as a central mediator of transforming growth factor (TGF) β signalling pathways.^10^ The role of the TGFβ pathway as a tumour promoter or suppressor at the cancer cell level is still a matter of debate, owing to its differential effects at the early and late stages of carcinogenesis. In contrast, at the microenvironment level, the TGFβ pathway contributes to generate a favourable microenvironment for tumour growth and metastasis throughout all the steps of carcinogenesis.^11^

The number of genetically altered driver genes in a PDAC varies widely, with <40% having an alteration in all four genes.^8^ Moreover, the number of driver gene alterations has been shown to be associated with overall survival, and patients with PDACs harbouring one to two driver alterations had the longest survival.^8,9^

A few other mutations (*KDM6A, RBM10* and *MLL3*) occur at a frequency of ~10%,^12^ and most other detected mutations occur at a rate of <5%.^7^ Furthermore, the frequency of targetable alterations, such as microsatellite instability, *BRCA2* mutations and the uncommon *KRASG12C* mutations, is low.^7,13,14^

### Molecular subtyping

Collisson et al.^15^ were the first to classify PDAC into subtypes based on gene expression. By examining expression data from human and mouse cell lines, they were able to identify three prognostic subtypes: classical, quasi-mesenchymal and exocrine-like. The classical subtype was defined by its high expression of adhesion-specific genes and epithelial genes, and conferred the best chance of survival. The quasi-mesenchymal subtype showed a higher expression of mesenchymal genes and was associated with the poorest prognosis. The exocrine subtype was reported to be associated with the expression of genes related to digestive enzymes, characteristic of the exocrine pancreatic function.

In 2015, Moffitt et al.,^16^ after incorporating expression microarray data from primary and metastatic tumours as well as normal samples, identified specific gene expression patterns that resulted in the identification of two tumour subtypes: a classical subtype that resembled the classical group of Collisson et al.^15^ and a ‘basal-like’ subtype with poor prognosis. Furthermore, they defined ‘normal’ and ‘activated’ stromal subtypes, which were independently prognostic.

In a more recent study, Bailey et al.,^12^ after the genomic analysis of 456 PDACs, identified 32 recurrently mutated genes from 10 pathways: KRAS, TGFβ, WNT, NOTCH, ROBO/SLIT signalling, G1–S transition, SWI-SNF, chromatin modification, DNA repair and RNA processing. Expression analysis yielded four subtypes of PDAC: squamous, pancreatic progenitor, immunogenic and aberrantly differentiated endocrine exocrine (ADEX). The squamous subtype described by Bailey et al.^12^ overlaps with the quasi-mesenchymal subgroup described by Collisson et al.^15^ and was associated with worse overall survival. Tumours of the squamous subtype were reported to be characterised by the presence of gene programmes and networks involved in the regulation of inflammation, hypoxia response and TGFβ signalling, among other roles, and showed upregulated expression of *TP63ΔN* along with frequent *TP53* mutations, as well as activation of epidermal growth factor signalling.^12^ Pancreatic progenitor tumours were defined by transcriptional networks containing transcription factors involved in early pancreatic development (e.g. FOXA2/3, PDX1 and MNX1). ADEX class (a subclass of pancreatic progenitor) displayed upregulation of genes that regulate networks involved in the later stages of pancreatic development and differentiation, such as KRAS activation and both exocrine (NR5A2 and RBPJL) and endocrine differentiation (NEUROD1 and NKX2-2). Immunogenic tumours were associated with significant immune infiltrate and contained upregulated immune networks including pathways involved in acquired immune suppression.

However, data from the TCGA pancreas cancer project^7^ support the existence of only two PDAC subtypes: the basal-like, alias squamous, alias quasi-mesenchymal subtype, which identifies PDACs with a poor prognosis and is characterised by basal markers, and the classical, alias pancreatic progenitor subtype, which is characterised by differentiated ductal markers and identifies PDACs with a better prognosis. Other subtypes, including the ADEX and the immunogenic subtypes, were found to exhibit low neoplastic cellularity, suggesting that stroma and normal pancreatic tissue might have greatly contributed to the molecular signatures of these subtypes.^7^

In accordance with the existence of two PDAC subtypes, Mueller et al.,^17^ after performing unbiased hierarchical clustering of RNA-sequencing data from mouse PDAC cell cultures, identified two clusters that largely overlapped with the two main human subtypes: C1, with ‘mesenchymal cell differentiation’, and C2, with ‘epithelial cell differentiation’. Their results linked the aggressive C1 PDAC subtype with the highest expression levels of *Kras*^G12D^ and Ras-related transcriptional programs. Moreover, screening of human transcriptome data revealed that undifferentiated human PDACs were characterised by a reduced expression of genes involved in ‘epithelial differentiation’ and a strong upregulation of gene sets enriched for epithelial–mesenchymal transition (EMT) and signalling downstream of Ras. Their results support the widespread effects of oncogenic dosage variation on cell morphology and plasticity, as well as clinical outcome, with the highest *Kras*^MUT^ levels underlying aggressive, undifferentiated phenotypes.^17^

Qian et al.^9^ also analysed protein expression and DNA alterations for the *KRAS, CDKN2A, SMAD4* and *TP53* genes by immunohistochemistry and next-generation sequencing in 365 resected PDACs, and reported that different patient outcomes were reflected in alterations in these four main driver genes. Thus, patients with *KRAS* mutant tumours had poorer disease-free survival compared with patients with *KRAS* wild-type tumours, whereas patients with a greater number of altered driver genes had poorer disease-free survival and overall survival than patients with a lower number of driver gene alterations.

Furthermore, key epigenetic pathways, most likely working as effectors of well-known genetic alterations, have been shown to influence the PDAC phenotype.^18^ It is thus likely that, at some moment during tumorigenesis, a combination of environmental and tumour-intrinsic factors, such as the TME, pushes cells through various epigenetic landscapes.^18^

The practical application of molecular results to guide patient selection for individual treatment is currently limited in patients with PDAC. However, several opportunities are emerging that might lead to the identification of novel therapeutic targets.

## Morphologic characteristics of pancreatic cancer

### Tumour budding, partial EMT and cancer stem cells

**Fig. 1:**
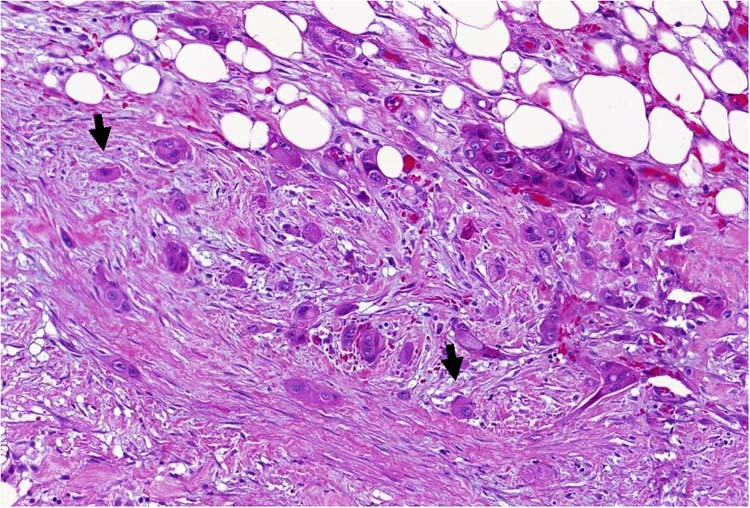
Haematoxylin/eosin-stained PDAC (× 400), with many tumour buds (arrows) and a microenvironment poor in immune cells

Although our understanding of the complex interplay between tumour cells and host cells in the TME is increasing, the specific role of the tumour cells in modifying their surroundings is still not well characterised. Aggressive PDACs display increased numbers of dissociative growing, migrating tumour cells at the invasive front, termed tumour buds, which have been proved to represent an independent adverse prognostic factor (Fig. 1).^19–23^ Moreover, it has been shown that the phenomenon of tumour budding is genetically very similar to the process of EMT, suggesting the existence of a partial EMT-like state.^24–26^ For example, tumour buds often show reduced E‑cadherin expression and loss of β‑catenin expression at the cell membrane, whereas they overexpress other EMT biomarkers such as zinc finger E‑box-binding homeobox proteins 1 and 2 (ZEB1 and ZEB2), Snail and N-cadherin.^24–27^ Furthermore, markers of apoptosis and proliferation are almost always absent in tumour buds, which confirms that migration and proliferation cannot take place simultaneously.^27^

The budding phenotype also appears to be influenced by microRNA dysregulation.^20^ Tumour buds display reduced expression of miR‑200b and miR‑200c, which correlates inversely with the increased expression of ZEB1 and ZEB2 in PDAC cases with high-grade budding.^20,28^ As members of the miRNA-200 family have been shown to exert strong suppressive effects on cell transformation, proliferation, migration, invasion, tumour growth and metastasis,^29–31^ their marked downregulation is likely to have a crucial role in the formation of tumour budding. The negative relationship between ZEB proteins and miRNA-200 family members has been described in various carcinomas and is considered to constitute the molecular basis for stabilisation of either the epithelial or the mesenchymal state in the context of EMT.^30^ This mechanism seems to be used by the tumour buds to establish their partial EMT-like state. There is also evidence that other components of the TME, such as the surrounding stromal cells, support EMT-like tumour budding by expressing high levels of E-cadherin suppressors and/or by contributing to the miRNA dysregulation. This highlights the role of the stroma in establishing an environment that is permissive to the presence of tumour buds.^20,24^

An increasing number of observations also link EMT to features of cancer stem cells,^32^ supporting the suggestion that tumour buds might represent a subpopulation of migrating cancer stem cells. WNT, which is strongly associated with the promotion of a stem cell-like phenotype,^33^ is also involved in the development of tumour budding.^24,34^ Furthermore, EMT-like cells (like tumour-budding cells) share similar molecular characteristics with cancer stem cells, as they are both drug resistant and have a high metastatic potential.^35^

Interestingly, recent studies of colorectal cancer have shown that tumour buds do not differ genetically from the other tumour cells. For example, none of the driver mutations was found to be exclusively present in the tumour buds and no new driver mutations were detected in the budding cells.^36^ These data further suggest that genetic alterations driving EMT features are an early phenomenon in tumorigenesis, whereas the EMT phenotype can be intensified through local factors derived from the TME.

Increasing awareness and understanding of tumour buds and their association with other cells, especially immune cells, in the TME, might lead to integrated prognostic factors and scores, which, by combining host-related and tumour-related factors, could eventually lead to better patient stratification and therapy guidance.

### PDAC subtypes and histological features

Comprehensive integrated genomic analysis of PDACs and their histopathological variants using a combination of whole-genome and deep-exome sequencing with gene copy number analysis and RNA expression profiles not only helped define four subtypes with survival differences and the different transcriptional networks that underpin them, but also showed associations between subtypes and distinct histopathologic characteristics.^12^ Thus, squamous subtype was associated with an increased number of carcinomas with adenosquamous morphology. Pancreatic progenitor and immunogenic subtypes showed frequently a mucinous non-cystic (colloid) morphology and mucinous adenocarcinomas arising from intraductal papillary mucinous neoplasms (IPMN) clustered with these subtypes. Furthermore, the ADEX subtype included rare acinar cell carcinomas.^12^

## Pancreatic cancer stroma and cancer-associated fibroblasts (CAFs)

PDAC microenvironment is characterised by a dense desmoplastic stroma, and CAFs are an important stromal cell population. Indeed, the stromal reaction accounts for up to 50-80% of the tumour volume.^37^ The role of CAFs in PDAC progression, especially in the induction of immunosuppression, has been contradictorily discussed in the literature. Although desmoplasia based on many studies is thought to confer biological aggressiveness, contributing to immune suppression and supporting further tumour growth,^38–40^ two recent studies have demonstrated that in mouse models with pancreatic cancer, targeting the stroma resulted in undifferentiated, aggressive pancreatic cancer, revealing a protective role by stroma.^41,42^

Recently, Puleo et al.^43^ identified a classification system based on gene expression analysis of formalin-fixed PDAC samples. In addition to the tumour components (basal-like and classical) that validated existing classifications,^16^ they identified four distinct stromal components (structural vascularised, activated, inflammatory and immune), reflecting the heterogeneity of the PDAC microenvironment. Confirming previous results by Moffitt et al.,^16^ they report that the stroma-activated (characterised by higher levels of fibroblasts) and the pure basal-like tumour subtypes both have low immune infiltrates and worse prognosis in comparison with the other subtypes.^43^ Thus there seems to exist a complex interplay between immune cell infiltration, stromal fibroblasts and tumour cells, leading to tumour-promoting or tumour-suppressing functions upon activation or abrogation of specific pathways.

## The immune landscape of pancreatic cancer

The role of the immune microenvironment of pancreatic cancer as an important prognostic/predictive feature is starting to emerge. PDAC is traditionally considered a ‘non-immunogenic’ neoplasm, and preclinical data support that pancreatic cancer can employ multiple means of immune evasion. Such mechanisms include the recruitment of regulatory immune cells, the secretion of immunosuppressive chemokines (such as stromal cell-derived factor 1, also known as CXC motif chemokine 12) and different cytokines (interleukin (IL)-1, IL-6, IL-10, TGFβ, tumour necrosis factor α and granulocyte–macrophage colony-stimulating factor), as well as the expression of cell-surface proteins, such as programmed death-ligand 1 (PD-L1), cytotoxic T-lymphocyte-associated protein 4 (CTLA4) and colony-stimulating factor 1 receptor (CSF1R). PD-L1 and CTLA4 are checkpoint inhibitor molecules, which confer inhibitory signals to the immune cells.^44,45^ PD-L1 has been reported to be overexpressed in PDACs, and this overexpression correlates with worse prognosis of the patients.^46^ CSF1R is located predominantly on myeloid cells and is involved in macrophage recruitment, its significance being underscored by the fact that macrophages are the dominant leucocyte population in human PDAC stroma, with an important functional contribution to the squamous PDAC subtype.^47^

### Tumour-associated macrophages (TAMs)

Regarding innate immunity, macrophages compose the most-dominant immune cell population in many tumours, including PDAC, and increased numbers of macrophages have been shown to correlate with poor prognosis.^12,46,47^ In autochthonous KPC PDAC mouse models, loss of macrophages in the TME, through CSF1R inhibition for example, was associated with tumour regression and T-cell activation, independently of PD-1 inhibition. Interestingly, it was also associated with downregulation of the squamous gene expression programmes and activation of ADEX and immunogenic gene programmes, leading to switch of PDAC subtypes.^47,48^

### T-cell heterogeneity

Although T cells are abundant in the stroma of human primary PDAC, and patients with higher levels of CD4+ and/or CD8+ T cells have significantly prolonged survival, most PDACs develop an immunosuppressive microenvironment that restricts the infiltration of anti-tumour T cells.^46,49^ In this regard, differential immune cell recruitment could be reflective of distinct immunosuppressive mechanisms. For example, the immune microenvironment of a large number of PDACs shows increased infiltrates of T-regulatory cells (Tregs) and TAMs with M2 polarisation, as well as myeloid-derived suppressive cells, which block the anti-tumour activities of the effector CD4+ and CD8+ T cells.^49–51^ Moreover, changes in the immunogenicity of cancer cells (so-called ‘immunoediting’) can lead to immune-resistant clones.^44^

On the other hand, there seems to exist a subgroup of immunogenic PDACs with a TME that is not only rich in effector CD4+ and CD8+ T cells but also poor in immunosuppressive immune cell populations, and this cell composition is associated with a better patient outcome.^49,52^ Moreover, a subset of these PDACs shows an increased number of peritumoural B lymphocytes, whereas the CD20+ and CD3+ stromal immune cell infiltrates can give rise to tertiary lymphoid tissue (TLT).^53^ The presence of TLT seems to convey a strong anti-tumour effect, resulting in favourable features and a survival advantage in PDAC.^52,53^

A recent study has shown that not only the relative abundance of T cells, but also the distribution and spatial relationship between T-cell subpopulations and cancer cells can give a more accurate picture of their biological interactions.^49^ Thus, the anti-tumour activity of cytotoxic T cells was particularly significant when these cells were found in the direct vicinity of cancer cells.^49^ Interestingly, although the desmoplastic stroma of PDAC has been hypothesised to hamper the anti-tumour T-cell response by sequestering T cells away from tumour cells,^54^ recent observations showing that PDACs that differ in their abundance of pericellular cytotoxic T-cell infiltrates do not differ in the levels of α-smooth muscle actin or collagen-I deposition questioned the impact of desmoplasia on the infiltration of cytotoxic T cells.^49^

### Neoantigens

Some recent studies have linked immune infiltration with neoantigen levels in cancer cells.^55,56^ However, other authors have suggested that, although this is mostly the case for cancers characterised by recurrent mutations, it does not hold for cancers driven by recurrent copy number alterations, such as pancreatic cancer.^57^ Interestingly, Balachandran et al.^55^ indicated that the neoantigen quality, rather than quantity, might modulate immunogenicity in PDAC, suggesting that the strong immune response against specific neoantigens generated during disease evolution, such as neoantigens in mucin 16, can lead to long-term survival.

Taken together, evidence from many studies supports the notion that PDAC immunity has a complex nature and predominantly comprises a heterogeneous T-cell population, whereas the interaction between immune and cancer cells creates a dynamic equilibrium that affects the progression of the disease. This underlines the growing need for diagnostic approaches addressing these microenvironmental signatures, enabling a sensitive and specific appraisal of the immune cell activity in PDAC.

## Integrated subtypes of pancreatic cancer

As discussed above, various high-throughput genomic and transcriptomic analyses have improved our ability to classify PDACs into distinct subtypes, and integrated classifications have begun to refine how we stratify this disease based on pathologic, genomic and immunologic criteria.

Evidence from many previous studies supports the presence of distinct PDAC subgroups with significant variation concerning their genetic background and the cellular composition of their microenvironment, resulting in different phenotypic and prognostic/predictive categories.^12,15,16,58^ Moreover, it seems that, within each PDAC subgroup, different mechanisms can shift the balance between the cancer cell and immune cell populations, thus creating a variety of ‘permissive’ conditions for tumour growth, emphasising the profound diversity in the nature of the immune response in PDAC. These subtypes represent various scenarios of tumour–host interactions, resulting in the emergence of different tumour mechanisms for evading the host immune response, with a subsequent impact on morphology and clinical behaviour.

### Immune-escape phenotype

**Fig. 2:**
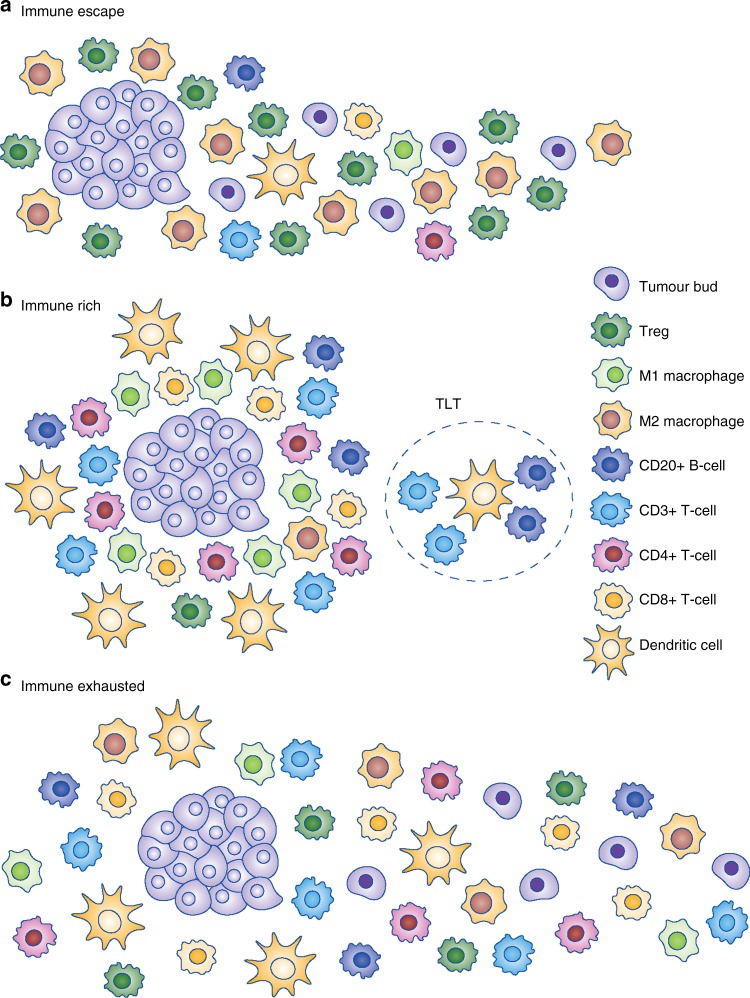
Schematic representation of the three main immunophenotypes of pancreatic cancer

**Fig. 3:**
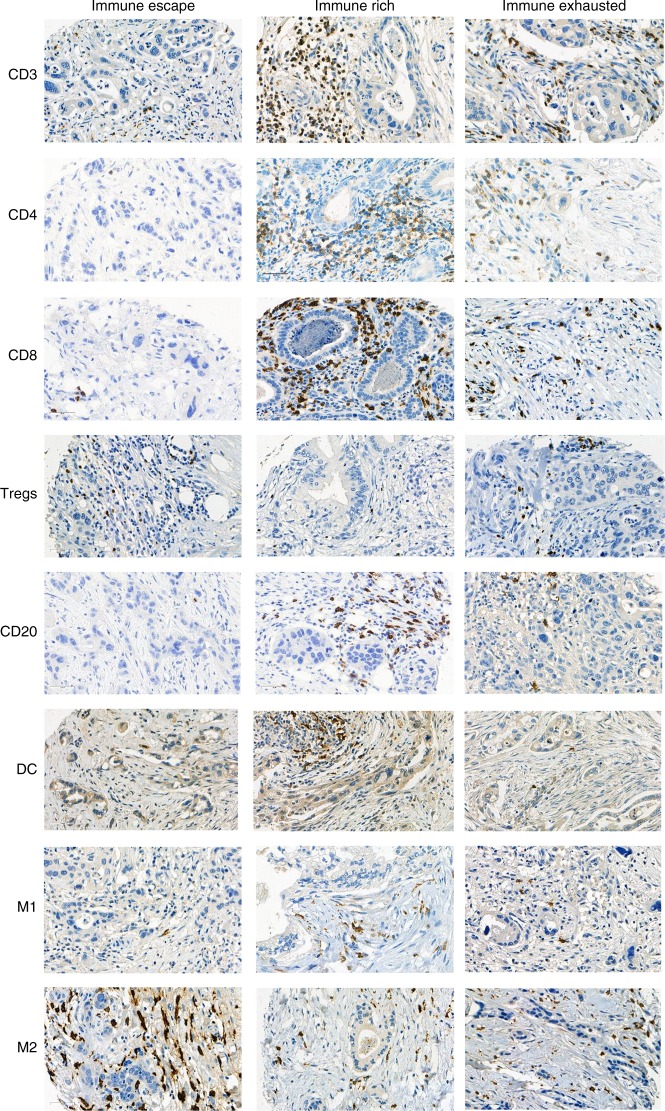
Immunohistochemical expression of the immune markers CD3, CD4, CD8, CD20, Foxp3 (Tregs), dendritic cells (DC), iNOS (M1) macrophages and CD163 (M2) macrophages across the immunophenotypes, × 400

**Fig. 4:**
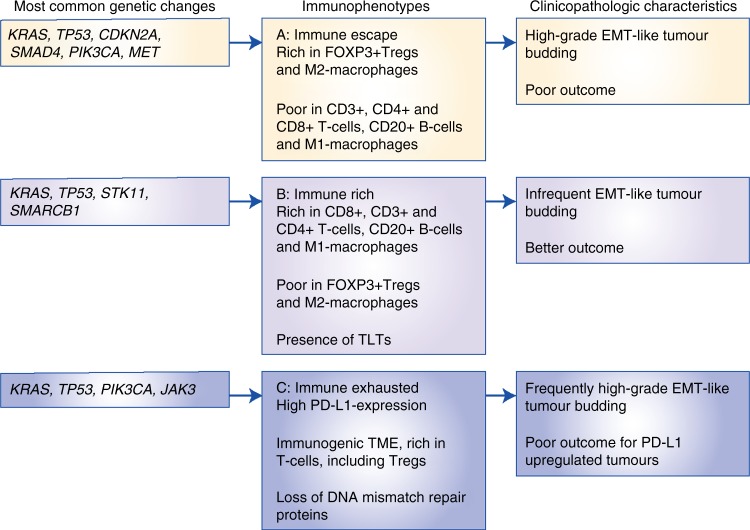
Summary of the main findings of the three different PDAC immunophenotypes

The first scenario encompasses the majority of PDACs that display highly immunosuppressive features, with a TME rich in Tregs and M2-polarised macrophages and poor in effector T cells, indicating an ‘immune-escape’ mechanism for evading the host immune response.^59^ These PDACs are, among others, characterised by an upregulation of EMT-promoting factors, including somatic genetic alterations and microRNA dysregulation, resulting in an aggressive phenotype with high-grade EMT-like tumour budding, unfavourable clinicopathologic features and poor prognosis (Figs. 2a, 3, 4, Immunophenotype A). This suggests that tumour-budding cells probably interact with, and are able to modulate, their microenvironment to create tumour-permissive conditions that guarantee their survival. The molecular and clinical characteristics of this phenotype show similarities with the squamous subtype described by Bailey et al.^12^ and/or the quasi-mesenchymal subtype described by Collisson et al.,^15^ as well as the ‘basal’ subtype by Moffitt et al.^16^ For example, tumours of the squamous subtype are characterised by gene programmes and networks involved in the regulation of epithelial cell plasticity and EMT.^12^ Some of these pathways are also directly involved in mechanisms that drive immune evasion and reduce immune cell infiltration.^60,61^

### Immune-rich phenotype

The second scenario applies to PDACs with highly cytotoxic immune phenotypes. The molecular and clinical pattern of these PDACs is more compatible with the pancreatic progenitor subtype outlined by Bailey et al.^12^ or the classical subgroup described by Collisson et al.^15^ and Moffitt et al.^16^ These PDACs are characterised by an ‘immune-rich’ microenvironment with high estimates of effector CD4+ and CD8+ T cells and M1 macrophages, and the frequent presence of TLTs along with reduced numbers of immunosuppressive immune cell populations such as Tregs and M2 macrophages. On a histomorphological level, they show low-grade tumour budding and favourable clinicopathological features associated with prolonged survival^52^ (Figs. 2b, 3, 4, Immunophenotype B). Tumours with highly cytotoxic microenvironments have been reported to be rich in immunogenic characteristics, such as an increased neoantigen load, and display a high mutational frequency in genes involved in the intrinsic DNA damage response or the upregulation of the antigen presentation machinery, and interferon signalling.^61^

### Mixed scenarios

The selective pressure of the highly cytotoxic immune phenotype might, however, result in the development of specific immune evasion mechanisms in some of these tumours.^61^ This could explain findings such as the unexpected presence of *ATM* mutations in PDACs with an immune-rich microenvironment.^52^ As the DNA damage response protein ATM is known to drive cytokine production, leading to increased recruitment of CD8+ cytotoxic T cells,^57^ abrogation of this function through mutation might represent an adaptive mechanism of the cancer cells in their effort to reduce immunotoxicity.

A further interesting example of an immune evasion mechanism is the one related to PD-L1 upregulation in the TME of a subset of PDACs. These PDACs show similarities to the immunogenic subtype described by Bailey et al.,^12^ and feature an ‘immune-exhausted’ microenvironment with unusual characteristics combining unfavourable clinicopathologic features such as frequent tumour budding along with an immune-rich microenvironment (Figs. 2c, 3, 4, Immunophenotype 3). It seems that, in these tumours, the selective pressure of the highly cytotoxic immune phenotype results in the development of mechanisms such as the amplification of the PD-L1/2 genes and/or the upregulation of inhibitory chemokines and of the JAK/STAT signalling pathway^62^ as a means of evasion from the host immune response. This indicates that, even though these tumours show immunophenotypic similarities to the PDACs, which have an ‘immune-rich’ microenvironment, the anti-tumour effect of the host immune response is literally cancelled by the evasive mechanisms, rendering the microenvironment tumour permissive and allowing for the formation of tumour buds, provoking a biological behaviour more similar to the PDACs with an immunosuppressive microenvironment.

In addition, there exists a very small PDAC subpopulation consisting of microsatellite unstable tumours that also exhibit an immunogenic microenvironment, almost half of them being carcinomas arising from IPMNs.^63^ Although mismatch repair (MMR) deficiency in PDAC is a rare event occurring at a very low frequency, it has, nevertheless, been correlated with clinical benefit after treatment with immune checkpoint blockade,^63,64^ so that testing for this deficiency in PDAC should be considered. Although both of the previous categories (i.e., PDACs with upregulation of PD-L1 and PDACs with MMR deficiency) could represent good candidates for the administration of checkpoint blockade therapy, the low frequency of these tumours might explain the limited success of this treatment modality in PDAC.

To summarise, an integrated approach of genomic/transcriptomic, clinicopathologic and immunophenotypic classification could help to guide the future development of immune and molecularly directed therapies in PDAC patients. This information may help define responsive subgroups for different treatment modalities and significantly expand the percentage of pancreatic cancer patients who will benefit from targeted therapeutic approaches by simultaneously excluding non-responders who will only have the adverse effects without the benefit of such therapy.

## Clinical opportunities

### Targeting mutations

Data from genome sequencing studies of resected PDAC indicate that PDAC lacks highly actionable simple somatic mutations.^12–14^ However, as molecular profiling of cancers is becoming more and more frequent, even a small number of actionable alterations might turn out to be important.

For example, germline or somatic mutations in one of the DNA damage repair genes *ATM, BRCA1, BRCA2* or *PALB2* could potentially sensitise these tumours to platinum-based chemotherapy or poly-(ADP-ribose) polymerase inhibition.^65^ In addition, there is a low prevalence of alterations in several genes that are potentially amenable to other targeted therapies, including *BRAF, PIK3CA, RNF43, STK11* and *JAK1*, as well as focal high-level amplifications in *ERBB2*.^7^

Other less-frequent molecular alterations likewise create opportunities for various treatment modalities towards a more individualised therapy approach. For example, MET pathway-targeted anticancer therapies might be effective in PDACs in which *MET* mutations are present, to additionally target EMT-like tumour budding.^66^ As mutations in the receptor tyrosine kinases MET and ERBB2 can be present simultaneously in a small number of PDACs,^67^ dual MET and ERBB inhibition, which has been shown to overcome intratumoural plasticity in osimertinib-resistant advanced non-small-cell lung cancer, could also be exploited in pancreatic cancer.^52^

### Immunotherapeutic approaches

The characteristics acquired by tumours in response to the surrounding immune infiltrates also open up new treatment strategies. Recent therapeutic modalities that involve taking advantage of some mechanisms of tumour immune evasion, such as the activation of immune checkpoints, have shown clinical success across a variety of cancer types.^68^ To date, however, single-agent immunotherapy trials have been unsuccessful in PDAC and other ‘non-immunogenic’ tumours, partly owing to the complex microenvironment that can considerably restrain immune cell infiltration and function. Immune checkpoint molecules, which transduce co-inhibitory signals to immunocompetent cells, are one of the most important components that confer an immunosuppressive capacity in the TME.^69^ As outlined above, CTLA4 and PD-1 are typical immune checkpoint molecules that are intimately involved in the suppression of anti-tumour immunity.^45^ In a mouse model of PDAC, it was shown that combining PD-1 and CTLA4 antagonists with myeloid growth factor receptor CSF1R blockade, which inhibits signalling by the CSF1R, elicited tumour regression, even in large established tumours. CSF1R blockade, however, upregulated T-cell checkpoint molecules, including PD-L1 and CTLA4, thereby restraining any beneficial therapeutic effects.^70^ Lately, attention has been given to reinforcing and restoring inadequate T-cell priming as a main cause of impaired immunogenicity and unresponsiveness to checkpoint inhibition. In this regard, CD40 activation has emerged as a promising factor for stimulating T-cell immunity and converting a non-immunogenic (cold) TME to an immunogenic (hot) one.^71^ CD40 is a cell-surface member of the tumour necrosis factor (TNF) receptor superfamily, most prominently expressed on dendritic cells (DCs), B cells and myeloid cells.^72^ Antigen-presenting cells, especially DCs, reinforced T-cell priming and function upon CD40 activation in a KPC mouse model of pancreatic cancer.^73^ Moreover, CD40 can drive the IL-12-dependent downregulation of PD-1 expression on T cells, reversing T-cell exhaustion and permitting tumour response in tumours that are otherwise refractory to PD-1 monoclonal antibodies.^74^ It thus seems that CD40 antibodies, especially in combination with chemotherapy, checkpoint inhibitor antibodies and other immune modulators, can confer T-cell-dependent anti-tumour activity.^71^ As CD40 is also expressed by B cells, it is possible that CD40 activation can additionally influence B-cell function in the TME, including B-regulatory cells and TLTs.^75^ For example, CD40-activated B cells are potent antigen-presenting cells, whereas it has been shown that the vaccination with antigen-loaded CD40-activated B cells induces a specific T-cell response in vivo comparable with that of DCs.^76^

### PDAC-derived organoids

More recently, combined genomic, transcriptomic and therapeutic profiling of patient-derived organoids was shown to be able to predict therapeutic responses to chemotherapy in both the adjuvant and advanced disease settings.^77^ A PDAC patient-derived organoid library has been generated, encompassing a broad spectrum of disease stages and facilitating the in-depth molecular characterisation of primary PDACs, thus bypassing the issue with frequently paucicellular PDAC specimens. Profiling of these organoids by using next-generation sequencing of DNA and RNA combined with pharmacotyping might help predict responses in PDAC patients and provide a rationale for prioritising certain therapeutic regimens.^77^

## Closing remarks

Categorisation of pancreatic cancer, integrating genomic, transcriptomic, immune-related and microenvironment-related factors, might facilitate the development of improved prognostic and theranostic biomarkers for this lethal disease. A meaningful next phase would be to expand the analysis of immunophenotypic, genetic and morphologic variables to allow for an even better association between histomorphologic findings, biological processes and signalling pathways in PDAC. This approach will lead to treatments with therapeutic modalities more precisely tailored to the unique disease biology of each patient in order to achieve improved outcomes. Based on the work of many researchers, one can be optimistic that such approaches will become a reality in the near future.

## Data Availability

Not applicable since this is a review study and not an original manuscript.
